# The development and validation of a scoring tool to predict the operative duration of elective laparoscopic cholecystectomy

**DOI:** 10.1007/s00464-018-6030-6

**Published:** 2018-01-16

**Authors:** Reshma Bharamgoudar, Aniket Sonsale, James Hodson, Ewen Griffiths, on behalf of the CholeS Study Group, West Midlands Research Collaborative, Ravinder S. Vohra, Amanda J. Kirkham, Sandro Pasquali, Paul Marriott, Marianne Johnstone, Philip Spreadborough, Derek Alderson, Ewen A. Griffiths, Stephen Fenwick, Mohamed Elmasry, Quentin M. Nunes, David Kennedy, Raja Basit Khan, Muhammad A. S. Khan, Conor J. Magee, Steven M. Jones, Denise Mason, Ciny P. Parappally, Pawan Mathur, Michael Saunders, Sara Jamel, Samer Ul Haque, Sara Zafar, Muhammad Hanif Shiwani, Nehemiah Samuel, Farooq Dar, Andrew Jackson, Bryony Lovett, Shiva Dindyal, Hannah Winter, Ted Fletcher, Saquib Rahman, Kevin Wheatley, Tom Nieto, Soofiyah Ayaani, Haney Youssef, Rajwinder S. Nijjar, Helen Watkin, David Naumann, Sophie Emesih, Piyush B. Sarmah, Kathryn Lee, Nikita Joji, Joel Lambert, Jonathan Heath, Rebecca L. Teasdale, Chamindri Weerasinghe, Paul J. Needham, Hannah Welbourn, Luke Forster, David Finch, Jane M. Blazeby, William Robb, Angus G. K. McNair, Alex Hrycaiczuk, Alexandros Charalabopoulos, Sritharan Kadirkamanathan, Cheuk-Bong Tang, Naga V. G. Jayanthi, Nigel Noor, Brian Dobbins, Andrew J. Cockbain, April Nilsen-Nunn, Jonathan de Siqueira, Mike Pellen, Jonathan B. Cowley, Wei-Min Ho, Victor Miu, Timothy J. White, Kathryn A. Hodgkins, Alison Kinghorn, Matthew G. Tutton, Yahya A. Al-Abed, Donald Menzies, Anwar Ahmad, Joanna Reed, Shabuddin Khan, David Monk, Louis J. Vitone, Ghulam Murtaza, Abraham Joel, Stephen Brennan, David Shier, Catherine Zhang, Thusidaran Yoganathan, Steven J. Robinson, Iain J. D. McCallum, Michael J. Jones, Mohammed Elsayed, Liz Tuck, John Wayman, Kate Carney, Somaiah Aroori, Kenneth B. Hosie, Adam Kimble, David M. Bunting, Kenneth B. Hosie, Adeshina S. Fawole, Mohammed Basheer, Rajiv V. Dave, Janahan Sarveswaran, Elinor Jones, Chris Kendal, Michael P. Tilston, Martin Gough, Tom Wallace, Shailendra Singh, Justine Downing Katherine A. Mockford, Eyad Issa, Nayab Shah, Neal Chauhan, Timothy R. Wilson, Amir Forouzanfar, Jonathan R. L. Wild, Emma Nofal, Catherine Bunnell, Khaliel Madbak, Sudhindra T. V. Rao, Laurence Devoto, Najaf Siddiqi, Zechan Khawaja, James C. Hewes, Laura Gould, Alice Chambers, Daniel Urriza Rodriguez, Gourab Sen, Stuart Robinson, Kate Carney, Francis Bartlett, David M. Rae, Thomas E. J. Stevenson, Kas Sarvananthan, Simon J. Dwerryhouse, Simon M. Higgs, Oliver J. Old, Thomas J. Hardy, Reena Shah, Steve T. Hornby, Ken Keogh, Lucinda Frank, Musallam Al-Akash, Emma A. Upchurch, Richard J. Frame, Michael Hughes, Clare Jelley, Simon Weaver, Sudipta Roy, Toritseju O. Sillo, Giorgios Galanopoulos, Tamzin Cuming, Pedro Cunha, Salim Tayeh, Sarantos Kaptanis, Mohamed Heshaishi, Abdalla Eisawi, Michael Abayomi, Wee Sing Ngu, Katie Fleming, Dalvir S. Bajwa, Vivek Chitre, Kamal Aryal, Paul Ferris, Michael Silva, Simon Lammy, Sarah Mohamed, Amir Khawaja, Adnan Hussain, Mudassar A. Ghazanfar, Maria Irene Bellini, Hamdi Ebdewi, Mohamed Elshaer, Gianpiero Gravante, Benjamin Drake, Arikoge Ogedegbe, Dipankar Mukherjee, Chanpreet Arhi, Lola Giwa, Nusrat Iqbal, Nicholas F. Watson, Smeer Kumar Aggarwal, Philippa Orchard, Eduardo Villatoro, Peter D. Willson, Kam Wa Jessica Mok, Thomas Woodman, Jean Deguara, Giuseppe Garcea, Benoy I. Babu, A. R. Dennison, Deep Malde, David Lloyd, Steve Satheesan, Omer Al-Taan, Alexander Boddy, John P. Slavin, Robert P. Jones, Laura Ballance, Stratos Gerakopoulos, Periyathambi Jambulingam, Sami Mansour, Naomi Sakai, Vikas Acharya, Mohammed M. Sadat, Lawen Karim, David Larkin, Khalid Amin, Amarah Khan, Jennifer Law, Saurabh Jamdar, Stella R. Smith, Keerthika Sampat, Kathryn M. O’shea, Mangta Manu, Fotini M. Asprou, Nabeela S. Malik, Jessica Chang, Marianne Johnstone, Michael Lewis, Geoffrey P. Roberts, Babu Karavadra, Evangelos Photi, James Hewes, Laura Gould, Alice Chambers, Dan Rodriguez, Derek A. O’Reilly, Anthony J. Rate, Hema Sekhar, Lucy T. Henderson, Benjamin Z. Starmer, Peter O. Coe, Sotonye Tolofari, Jenifer Barrie, Gareth Bashir, Jake Sloane, Suroosh Madanipour, Constantine Halkias, Alexander E. J. Trevatt, David W. Borowski, Jane Hornsby, Michael J. Courtney, Suvi Virupaksha, Keith Seymour, Sarah Robinson, Helen Hawkins, Sadiq Bawa, Paul V. Gallagher, Alistair Reid, Peter Wood, J. G. Finch, J. Guy Finch, J. Parmar, E. Stirland, James Gardner-Thorpe, Ahmed Al-Muhktar, Mark Peterson, Ali Majeed, Farrukh M. Bajwa, Jack Martin, Alfred Choy, Andrew Tsang, Naresh Pore, David R. Andrew, Waleed Al-Khyatt, Christopher Taylor, Santosh Bhandari, Adam Chambers, Dhivya Subramanium, Simon K. C. Toh, Nicholas C. Carter, Sophie Tate, Belinda Pearce, Denise Wainwright, Stuart J. Mercer, Benjamin Knight, Vardhini Vijay, Swethan Alagaratnam, Sidhartha Sinha, Shahab Khan, Shamsi S. El-Hasani, Abdulzahra A. Hussain, Vish Bhattacharya, Nisheeth Kansal, Tani Fasih, Claire Jackson, Midhat N. Siddiqui, Imran A. Chishti, Imogen J. Fordham, Zohaib Siddiqui, Harald Bausbacher, Ileana Geogloma, Kabita Gurung, George Tsavellas, Pradeep Basynat, Ashish Kiran Shrestha, Sanjoy Basu, Alok Chhabra, Mohan Harilingam, Mohamed Rabie, Mansoor Akhtar, Pradeep Kumar, Sadaf F. Jafferbhoy, Najam Hussain, Soulat Raza, Manzarul Haque, Imran Alam, Rabiya Aseem, Shakira Patel, Mehek Asad, Michael I. Booth, William R. Ball, Christopher P. J. Wood, Ana C. Pinho-Gomes, Ambareen Kausar, Moh’d Rami Obeidallah, Joseph Varghase, Joshil Lodhia, Donal Bradley, Carla Rengifo, David Lindsay, Sivakumar Gopalswamy, Ian Finlay, Stacy Wardle, Naomi Bullen, Syed Yusuf Iftikhar, Altaf Awan, Javed Ahmed, Paul Leeder, Guiseppe Fusai, Giles Bond-Smith, Alicja Psica, Yogesh Puri, David Hou, Fergus Noble, Karoly Szentpali, Jack Broadhurst, Ravindra Date, Martin R. Hossack, Yan Li Goh, Paul Turner, Vinutha Shetty, Manel Riera, Christina A. W. Macano, Anisha Sukha, Shaun R. Preston, Jennifer R. Hoban, Daniel J. Puntis, Sophie V. Williams, Richard Krysztopik, James Kynaston, Jeremy Batt, Matthew Doe, Andrzej Goscimski, Gareth H. Jones, Stella R. Smith, Claire Hall, Nick Carty, Jamil Ahmed, Sofoklis Panteleimonitis, Rohan T. Gunasekera, Andrea R. G. Sheel, Hannah Lennon, Caroline Hindley, Marcus Reddy, Ross Kenny, Natalie Elkheir, Emma R. McGlone, Rajasundaram Rajaganeshan, Kate Hancorn, Anita Hargreaves, Raj Prasad, David A. Longbotham, Dhakshinamoorthy Vijayanand, Imeshi Wijetunga, Paul Ziprin, Christopher R. Nicolay, Geoffrey Yeldham, Edward Read, James A. Gossage, Rachel C. Rolph, Husam Ebied, Manraj Phull, Mohammad A. Khan, Matthew Popplewell, Dimitrios Kyriakidis, Anwar Hussain, Natasha Henley, Jessica R. Packer, Laura Derbyshire, Jonathan Porter, Shaun Appleton, Marwan Farouk, Melvinder Basra, Neil A. Jennings, Shahda Ali, Venkatesh Kanakala, Haythem Ali, Risha Lane, Richard Dickson-Lowe, Prizzi Zarsadias, Darius Mirza, Sonia Puig, Khalid Al Amari, Deepak Vijayan, Robert Sutcliffe, Ravi Marudanayagam, Zayed Hamady, Abheesh R. Prasad, Abhilasha Patel, Damien Durkin, Parminder Kaur, Laura Bowen, James P. Byrne, Katherine L. Pearson, Theo G. Delisle, James Davies, Mark A. Tomlinson, Michelle A. Johnpulle, Corinna Slawinski, Andrew Macdonald, James Nicholson, Katy Newton, James Mbuvi, Ansar Farooq, Bhavani Sidhartha Mothe, Zakhi Zafrani, Daniel Brett, James Francombe, Philip Spreadborough, James Barnes, Melanie Cheung, Ahmed Z. Al-Bahrani, Giuseppe Preziosi, Tomas Urbonas, Justin Alberts, Mekhlola Mallik, Krashna Patel, Ashvina Segaran, Triantafyllos Doulias, Pratik A. Sufi, Caroline Yao, Sarah Pollock, Antonio Manzelli, Saj Wajed, Michail Kourkulos, Roberto Pezzuto, Martin Wadley, Emma Hamilton, Shameen Jaunoo, Robert Padwick, Mazin Sayegh, Richard C. Newton, Madhusoodhana Hebbar, Sameh F. Farag, John Spearman, Mohammed F. Hamdan, Conrad D’Costa, Christine Blane, Mathew Giles, Mark B. Peter, Natalie A. Hirst, Tanvir Hossain, Arslan Pannu, Yesar El-Dhuwaib, Tamsin E. M. Morrison, Greg W. Taylor, Ronald L. E. Thompson, Ken McCune, Paula Loughlin, Roger Lawther, Colman K. Byrnes, Duncan J. Simpson, Abi Mawhinney, Conor Warren, Damian McKay, Colin McIlmunn, Serena Martin, Matthew MacArtney, Tom Diamond, Phil Davey, Claire Jones, Joshua M. Clements, Ruairi Digney, Wei Ming Chan, Stephen McCain, Sadaf Gull, Adam Janeczko, Emmet Dorrian, Andrew Harris, Suzanne Dawson, Dorothy Johnston, Barry McAree, Essam Ghareeb, George Thomas, Martin Connelly, Stephen McKenzie, Krzysztos Cieplucha, Gary Spence, William Campbell, Gareth Hooks, Neil Bradley, Arnold D. K. Hill, John T. Cassidy, Michael Boland, Paul Burke, Deirdre M. Nally, Arnold D. K. Hill, Elmoataz Khogali, Wael Shabo, Edrin Iskandar, Gerry P. McEntee, Maeve A. O’Neill, Colin Peirce, Emma M. Lyons, Adrian W. O’Sullivan, Rohan Thakkar, Paul Carroll, Ivan Ivanovski, Paul Balfe, Matthew Lee, Des C. Winter, Michael E. Kelly, Emir Hoti, Donal Maguire, Priyadarssini Karunakaran, Justin G. Geoghegan, Frank McDermott, Sean T. Martin, Keith S. Cross, Fiachra Cooke, Saquib Zeeshan, James O. Murphy, Ken Mealy, Helen M. Mohan, Yuwaraja Nedujchelyn, Muhammad Fahad Ullah, Irfan Ahmed, Francesco Giovinazzo, James Milburn, Sarah Prince, Eleanor Brooke, Joanna Buchan, Ahmed M. Khalil, Elizabeth M. Vaughan, Michael I. Ramage, Roland C. Aldridge, Simon Gibson, Gary A. Nicholson, David G. Vass, Alan J. Grant, David J. Holroyd, M. Angharad Jones, Cherith M. L. R. Sutton, Patrick O’Dwyer, Frida Nilsson, Beatrix Weber, Tracey K. Williamson, Kushik Lalla, Alice Bryant, C. Ross Carter, Craig R. Forrest, David I. Hunter, Ahmad H. Nassar, Mavis N. Orizu, Katrina Knight, Haitham Qandeel, Stuart Suttie, Rowena Belding, Andrew McClarey, Alan T. Boyd, Graeme J. K. Guthrie, Pei J. Lim, Andreas Luhmann, Angus J. M. Watson, Colin H. Richards, Laura Nicol, Marta Madurska, Ewen Harrison, Kathryn M. Boyce, Amanda Roebuck, Graeme Ferguson, Pradeep Pati, Michael S. J. Wilson, Faith Dalgaty, Laura Fothergill, Peter J. Driscoll, Kirsty L. Mozolowski, Victoria Banwell, Stephen P. Bennett, Paul N. Rogers, Brendan L. Skelly, Claire L. Rutherford, Ahmed K. Mirza, Taha Lazim, Henry C. C. Lim, Diana Duke, Talat Ahmed, William D. Beasley, Marc D. Wilkinson, Geta Maharaj, Cathy Malcolm, Timothy H. Brown, Bilal Al-Sarireh, Guy M. Shingler, Nicholas Mowbray, Rami Radwan, Paul Morcous, Simon Wood, Abbas Kadhim, Duncan J. Stewart, Andrew L. Baker, Nicola Tanner, Hrishikesh Shenoy, Shazia Hafiz, Joshua A. De Marchi, Deepak Singh-Ranger, Elzanati Hisham, Paul Ainley, Stephen O’Neill, John Terrace, Sara Napetti, Benjamin Hopwood, Thomas Rhys, Justine Downing, Sam Kanavati, Maria Coats, Danail Aleksandrov, Charlotte Kallaway, Salama Yahya, Beatrix Weber, Alexa Templeton, Martin Trotter, Christina Lo, Ajit Dhillon, Nick Heywood, Yousif Aawsaj, Alhafidz Hamdan, Obuobi Reece-Bolton, Andrew McGuigan, Yousef Shahin, Aymon, Ali Alison Luther, James A. Nicholson, Ilayaraja Rajendran, Matthew Boal, Judith Ritchie

**Affiliations:** 1 College of Medical & Dental Sciences, University of Birmingham, Birmingham, UK; 2 Institute of Translational Medicine, University Hospitals Birmingham NHS Foundation Trust, Birmingham, UK; 3 Institute of Cancer and Genomic Sciences, College of Medical and Dental Sciences, University of Birmingham, Birmingham, UK; 4 Department of Upper Gastrointestinal Surgery, University Hospitals Birmingham NHS Foundation Trust, Birmingham, UK

**Keywords:** Laparoscopic cholecystectomy, Patient factors, Operative duration, Scoring tool, Prediction, Theatre utilisation

## Abstract

**Background:**

The ability to accurately predict operative duration has the potential to optimise theatre efficiency and utilisation, thus reducing costs and increasing staff and patient satisfaction. With laparoscopic cholecystectomy being one of the most commonly performed procedures worldwide, a tool to predict operative duration could be extremely beneficial to healthcare organisations.

**Methods:**

Data collected from the CholeS study on patients undergoing cholecystectomy in UK and Irish hospitals between 04/2014 and 05/2014 were used to study operative duration. A multivariable binary logistic regression model was produced in order to identify significant independent predictors of long (> 90 min) operations. The resulting model was converted to a risk score, which was subsequently validated on second cohort of patients using ROC curves.

**Results:**

After exclusions, data were available for 7227 patients in the derivation (CholeS) cohort. The median operative duration was 60 min (interquartile range 45–85), with 17.7% of operations lasting longer than 90 min. Ten factors were found to be significant independent predictors of operative durations > 90 min, including ASA, age, previous surgical admissions, BMI, gallbladder wall thickness and CBD diameter. A risk score was then produced from these factors, and applied to a cohort of 2405 patients from a tertiary centre for external validation. This returned an area under the ROC curve of 0.708 (SE = 0.013, *p* < 0.001), with the proportions of operations lasting > 90 min increasing more than eightfold from 5.1 to 41.8% in the extremes of the score.

**Conclusion:**

The scoring tool produced in this study was found to be significantly predictive of long operative durations on validation in an external cohort. As such, the tool may have the potential to enable organisations to better organise theatre lists and deliver greater efficiencies in care.

There are 70,000 cholecystectomies performed in the UK each year, making it one of the most common general surgical operations [1]. The average operative duration for this laparoscopic procedure is usually < 1 h [2]. With the average hourly cost for an operating theatre being £1200, efforts to utilise every minute of allocated theatre time is vital [3]. This is especially so in a resource constrained National Health Service (NHS) working environment that is required to save £20 bn by 2020 to remain sustainable for the future [4]. Poor planning can lead to cancellations, which are expensive for the Trust and, more importantly, distressing for patients. One particular study found that 63% of on-the-day cancellations were due to a lack of theatre time [5], with another study demonstrating that approximately 30% of lists are under-run, leaving the operating theatre idle [6]. Careful planning and scheduling is therefore paramount to increase operating theatre efficiency and, in doing so, it is estimated that NHS trusts can make efficiency savings of approximately £4 m per year [3]. In a time where rising demographic pressures are demanding ever increasing spending on healthcare, it is vital we optimise the use of our existing resources.

Previous research in other surgical areas has assessed a variety of patient factors and their impact on operative duration [7–11], but studies in laparoscopic cholecystectomy are few and a predictive scoring tool is yet to be developed. Traditionally, surgeons have estimated their operative durations, but research has demonstrated that these estimates are often inaccurate [6]. Many hospitals have now moved to electronic systems and central schedulers with the aim of reducing costs. However, such systems require significant learning time to adapt to organisational needs [12]. Historical procedure and surgeon data have been used to estimate operative duration. However, this is known to be of low accuracy, as it fails to account for pre-operative patient factors [12].

We aimed to create a clinically useful scoring tool to predict the operative duration of laparoscopic cholecystectomy using pre-operative patient factors and to externally validate its reliability using a separate dataset.

## Methods

Two datasets were used in this study, to allow for a risk score to be produced and externally validated:

### CholeS dataset

The CholeS study was a multicentre, prospective population-based cohort study that assessed variations in patient factors with outcomes of cholecystectomy [13, 14]. The protocol for this study has been published previously [15]. Data were collected from 8820 patients who underwent a laparoscopic cholecystectomy at 166 hospitals in the UK between March to April 2014, and was found to be 99.2% accurate by independent data validation. Pre-operative variables included patient demographics, indications for surgery, admission type, ASA grade, ultrasound findings and pre-operative ERCP. Surgical duration was calculated from the time (minutes) of skin incision to end of skin closure.

For this study, all patients undergoing emergency cholecystectomy were excluded (*N* = 1420), as these are associated with long procedure times and elective theatre utilisation was the focus of this study. In addition, those where the operative duration was not recorded (*N* = 168) were excluded, as were those with an operative duration < 10 min (*N* = 5), as these were thought to be unrealistic. This left a total of 7227 patients for analysis.

### Validation dataset

The validation dataset was retrospectively collected from the University Hospitals Birmingham NHS Foundation Trust (UHB)—a large tertiary hospital with 1213 inpatient beds and 32 operating theatres [16]. Data were collected for all elective laparoscopic cholecystectomies carried out between 2010 and 2016, excluding emergencies, as well as those having combined procedures. This left a total of *N* = 2405 patients for analysis. Data for surgical duration and pre-operative factors were gathered from a variety of electronic hospital systems, including the Lorenzo patient information system, Galaxy operating theatre system and PICS (Prescribing Information and Communication system). ASA grades were calculated using comorbidities derived from the informatics database, and were based on the definitions provided by the American Society of Anaesthesiologists [17].

### Statistical methods

Initially, the operative duration was dichotomised into groups of ≤ 90 vs. > 90 min. Univariable analyses were then performed, comparing the rates of operative durations > 90 min across a range of factors. Comparisons across categorical factors were made using Fisher’s exact tests, whilst Mann–Whitney tests were used to compare ordinal factors between the operative duration groups.

A multivariable analysis was then performed to identify independent predictors of operative duration. A binary logistic regression model was produced, with a forwards stepwise approach used to select variables for inclusion. The resulting model was then converted to a risk score, by rounding the beta coefficient (log-odds) of each factor to the nearest 0.5, after multiplying by a constant to minimise the impact of rounding errors. Where this resulted in negative values, the reference category was changed, such that all values in the score were positive. The predictive accuracy of the model was then assessed using ROC curves. The model was also applied to a second cohort of patients for external validation.

All analyses were performed using IBM SPSS 22 (IBM Corp. Armonk, NY), with *p* < 0.05 deemed to be indicative of statistical significance throughout.

## Results

### Demographics

After exclusions, data were available for 7227 surgeries in the derivation (CholeS) cohort. The patients had a mean age of 51 years (SD = 16), and the majority were female (74.8%). The median operative duration was 60 min, with an interquartile range (IQR) of 45–85 min. Operations lasted for > 90 min in 17.7% (*N* = 1279) of the cohort.

### Risk score derivation

Associations between the operative duration and a range of demographic and pre-operative factors are reported in Table 1. All of the factors considered were found to be significantly associated with longer operative durations. For this reason, a multivariable analysis was performed, in order to identify which factors were independently associated with operative duration (Table 2). This analysis found the likelihood of an operation taking > 90 min to increase significantly with BMI (*p* < 0.001), ASA grade (*p* < 0.001), and the number of previous surgical admissions that the patient had (*p* = 0.005). In addition, the indication on admission significantly influenced operative duration (*p* < 0.001), with patients admitted with acalculous and cholecystitis being the most likely to have operations lasting > 90 min. Patients with a thickened gallbladder, a dilated CBD diameter, or who had received a pre-operative CT or planned intra-operative cholangiogram were significantly more likely to take > 90 min (all *p* < 0.001). In addition, patients of male gender (*p* = 0.002) and aged 40+ (*p* = 0.004) were also at significantly higher risk of requiring a longer operative duration.

**Table 1:** Univariable analysis of associations between operative duration and both demographic and pre-operative factors

	*N*	Operative duration > 90 min	*p* Value
Age (years)			< 0.001**
< 30	854	90 (10.5%)	
30–39	1035	128 (12.4%)	
40–49	1397	237 (17.0%)	
50–59	1534	297 (19.4%)	
60–69	1381	282 (20.4%)	
70+	1023	245 (23.9%)	
Gender			< **0.001**
Female	5406	852 (15.8%)	
Male	1821	427 (23.4%)	
Indication for surgery			< **0.001**
Acalculous/cholecystitis	1739	483 (27.8%)	
CBD stone	479	145 (30.3%)	
Colic/dyskinesia/polyp	4435	541 (12.2%)	
Pancreatitis	570	109 (19.1%)	
BMI			< 0.001*
<25	1475	219 (14.8%)	
25–30	2465	424 (17.2%)	
31–35	1689	305 (18.1%)	
>35	1324	279 (21.1%)	
CBD diameter			< **0.001**
Normal	6013	936 (15.6%)	
Dilated	1063	310 (29.2%)	
Gallbladder wall			< **0.001**
Normal	5017	708 (14.1%)	
Thick walled	2053	530 (25.8%)	
Pre-operative MRCP			< **0.001**
No	5325	857 (16.1%)	
Yes	1819	419 (23.0%)	
Pre-operative CT			< **0.001**
No	6158	1006 (16.3%)	
Yes	978	271 (27.7%)	
Pre-operative ERCP			< **0.001**
No	6349	1049 (16.5%)	
Yes	783	227 (29.0%)	
Grade of senior surgeon			0.005*
<ST5	308	42 (13.6%)	
>ST6	1165	183 (15.7%)	
Consultant	5748	1052 (18.3%)	
Planned intra-op cholangiogram			< **0.001**
No	6519	1070 (16.4%)	
Yes	655	203 (31.0%)	
Number of previous surgical admissions			< 0.001*
0	4006	535 (13.4%)	
1	2424	546 (22.5%)	
2	486	118 (24.3%)	
>2	170	57 (33.5%)	
ASA			< 0.001*
1	2803	352 (12.6%)	
2	3687	722 (19.6%)	
>2	690	199 (28.8%)	

Data reported as *N* (%), with *p* values from Fisher’s exact tests, unless stated otherwise

*p Value from a Mann–Whitney test, to account for the ordinal nature of the factor

**p Value from a Mann–Whitney test, using the exact age. Bold *p* values are significant at *p* < 0.05

**Table 2:** Multivariable analysis of predictors of > 90 min operations

	Beta^a^	Odds ratio (95% CI)	*p* Value
Age (years)			**0.004**
<30	0	1	–
30–39	0.058	1.06 (0.78–1.45)	0.714
40–49	0.430	1.54 (1.16–2.05)	**0.003**
50–59	0.438	1.55 (1.17–2.06)	**0.002**
60–69	0.366	1.44 (1.07–1.93)	**0.015**
70+	0.380	1.46 (1.07–2.00)	**0.017**
Gender (male)	0.241	1.27 (1.09–1.48)	**0.002**
Indication			< **0.001**
Acalculous/cholecystitis	0	1	*–*
CBD stone	− 0.154	0.86 (0.66–1.12)	0.258
Colic/dyskinesia/polyp	− 0.527	0.59 (0.49–0.71)	< **0.001**
Pancreatitis	− 0.627	0.53 (0.41–0.70)	< **0.001**
BMI			< **0.001**
<25	0	1	*–*
25–30	0.208	1.23 (1.01–1.49)	**0.035**
31–35	0.291	1.34 (1.09–1.65)	**0.006**
>35	0.532	1.70 (1.36–2.13)	< **0.001**
CBD diameter (dilated)	0.535	1.71 (1.42–2.05)	< **0.001**
Gallbladder wall (thick)	0.371	1.45 (1.24–1.70)	< **0.001**
Pre-operative CT	0.320	1.38 (1.15–1.65)	< **0.001**
Planned intra-op cholangiogram	0.706	2.03 (1.66–2.47)	< **0.001**
Number of previous surgical admissions			**0.005**
0	0	1	*–*
1	0.202	1.22 (1.03–1.45)	**0.020**
2	0.227	1.25 (0.96–1.64)	0.095
>2	0.630	1.88 (1.29–2.74)	**0.001**
ASA			< **0.001**
1	0	1	*–*
2	0.225	1.25 (1.06–1.47)	**0.007**
>2	0.630	1.88 (1.48–2.39)	< **0.001**

Results are from a multivariable binary logistic regression model with a forward stepwise approach to variable selection. All factors from Table 1 were considered for inclusion in the model. Bold *p* values are significant at *p* < 0.05

^a^The beta coefficients (i.e. log-odds) from the model

These 10 factors were then combined to form a risk score (Table 3). This score has a potential range from 0 to 20, although the observed range in the cohort was 0.5–17.5 (median = 5). A ROC curve analysis returned an area under the curve (AUROC) of 0.696 (SE = 0.009, *p* < 0.001) for the prediction of operations lasting > 90 min.

**Table 3:** Risk score

	Points
Age (years)	
<40	0
40+	1.5
Gender (male)	
Female	0
Male	1
Indication	
Pancreatitis	0
Colic/dyskinesia/polyp	0.5
CBD stone	2
Acalculous/cholecystitis	2.5
BMI	
<25	0
25–35	1
>35	2
CBD diameter	
Normal	0
Dilated	2
Gallbladder wall	
Normal	0
Thick	1.5
Pre-operative CT	
No	0
Yes	1.5
Planned intra-op cholangiogram	
No	0
Yes	3
Number of previous surgical admissions	
0	0
1–2	1
>2	2.5
ASA	
1	0
2	1
>2	2.5

Based on the multivariable analysis in Table 2. The number of points for each factor was calculated by rounding the beta coefficient to the nearest 0.5, after multiplying by 4 to minimise rounding errors. Categories for a factor that had the same number of points (e.g. age < 30 and 30–39) were combined to simplify the table

### Validation

**Fig. 1:**
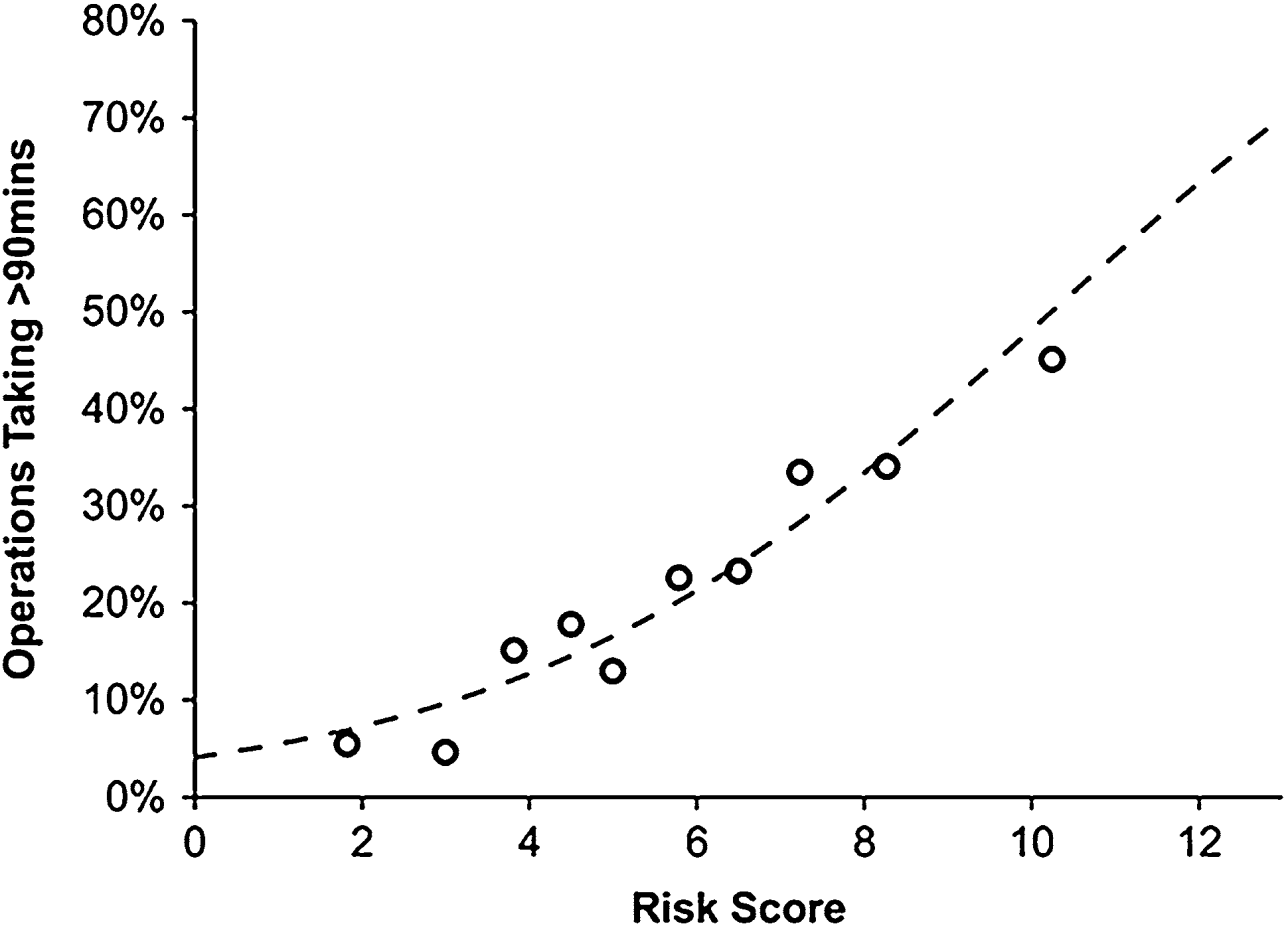
Demonstrates the relationship between the risk score and the proportion of operations taking > 90 min in the validation cohort. Of the 470 patients with risk scores of 0–3, only 5.1% (*N* = 24) of operations took > 90 min, increasing to 41.8% (109/261) in those with risk scores > 8

The risk score was then applied to a cohort of *N* = 2405 patients from UHB for external validation. This cohort had a similar operative duration to the derivation cohort, with a median of 66 min (IQR: 52–85), and with 20.0% (*N* = 481) of operations taking > 90 min. The median risk score was found to be 4.5, with a range from 0 to 14.5. The score as a whole had a similar degree of predictive accuracy to that observed in the derivation cohort, with an AUROC of 0.708 (SE = 0.013, *p* < 0.001) (Fig. 1).

## Discussion

Our study has created a scoring tool that uses pre-operative patient factors to predict the probability that a laparoscopic cholecystectomy will take > 90 min. This scoring tool has also been successfully externally validated against a separate dataset and has demonstrated predictive accuracy. The results showed that, for low-scoring individuals vs. high-scoring individuals, the proportion of operations taking > 90 min increases significantly from 5.8 to 41.4%.

We hope this scoring tool could enable better planning and utilisation of elective theatre lists. A better understanding of patient factors that result in increased operative duration and how this affects surgeon workload can help to optimise theatre scheduling and result in fewer cancellations. With theatres being one of the most expensive resources to run [18], even small improvements in theatre utilisation have the potential to impact costs greatly, and with many trusts under pressure to tackle unsustainable deficits [19], it is possible that utilising this scoring tool may be helpful in addressing this issue. This is particularly possible, given that laparoscopic cholecystectomies are one of the most commonly performed operations in the NHS [1].

Thiels et al. [12] assessed the surgical duration of 1801 elective laparoscopic cholecystectomies from 2007 to 2013 and found female sex, BMI, ASA grade and pre-operative laboratory results to be predictive factors in influencing operative duration. They used a large group of patients from the NSQIP (American College of Surgeons National Surgical Quality Improvement Program) to validate their findings [12]. Zdichavsky et al. [20] performed a retrospective analysis of 677 consecutive patients undergoing laparoscopic cholecystectomies from 2004 to 2007 (excluding conversions, intra-operative cholangiogram and concurrent liver cirrhosis) and found male sex, obesity, acute cholecystitis and previous abdominal surgery to be independently predictive of duration. In a small study of only 138 cholecystectomies, junior residents took significantly longer to complete a cholecystectomy than their senior counterparts (*p* < 0.05) [21].

In our study, factors found to be independently predictive of operative duration were patient age, gender, ASA grade, operative indication, BMI, CBD diameter, gallbladder wall thickness, pre-operative CT scan, planned intra-operative cholangiogram and the number of previous surgical admissions. These factors are broadly similar to previous work by other researchers [12, 20, 21]. Our study expands on their work and has developed a clinically useful scoring tool. To our knowledge, this is the first study to use patient factors to create a validated scoring tool to predict operative duration for elective laparoscopic cholecystectomies.

Our study uses high-quality, validated, prospective data that were collected as part of the CholeS study. The substantial cohort of 7227 patients is considerably larger than those used by past researchers, which altogether gives greater assurance as to the reliability of the derived scoring tool. In addition, we have external validated our scoring tool and its utility to successfully predict operative duration. Our scoring tool can be used pre-operatively and was developed in a dataset which included patients who underwent conversion to open surgery and cholangiography and is therefore more generalizable. There are however some limitations that should be considered when analysing this study’s results. The CholeS study did not collect data on pre-operative blood results, such as white cell count or CRP, or whether the patient had previous Upper GI surgery, which may indicate difficult surgery [22]. We recognise that the validation dataset was retrospectively collected from routine hospital data and therefore may have some inaccuracies. ASA grades were calculated retrospectively based on information from the patients’ clinical records. However, even when ASA is calculated by anaesthetists there is an element of bias and variation [23]. Furthermore, this scoring tool has been developed for elective cholecystectomy data and should not be used to predict the duration of acute operations.

## Conclusion

We have created a scoring tool to predict operative durations of elective laparoscopic cholecystectomies using pre-operative patient factors. Whilst previous research may have examined the significance of individual factors, there remained a lack of a formal scoring tool. Using the 7227 patient CholeS dataset to derive the scoring system, and a UHB database of 2405 patients to subsequently validate the tool, we have shown that it is possible to predict operations that are likely to last greater than 90 min. This could be useful for theatre schedulers to ensure theatre lists are planned appropriately to optimise theatre utilisation and achieve cost savings. For example, the tool could be used to select the most appropriate patients to fit into a half day operating list with a low risk of overrunning. Another use could be to place patients with a long operative duration (and hence higher operative difficultly) on a specialist surgeon’s operating list.
